# Chiral Orientation of Skeletal Muscle Cells Requires Rigid Substrate

**DOI:** 10.3390/mi8060181

**Published:** 2017-06-08

**Authors:** Ninghao Zhu, Hoi Kwan Kwong, Yuanye Bao, Ting-Hsuan Chen

**Affiliations:** 1 Department of Mechanical and Biomedical Engineering, City University of Hong Kong, 83 Tat Chee Avenue, Hong Kong, China; ninghazhu2-c@my.cityu.edu.hk (N.Z.); hoikkwong2-c@my.cityu.edu.hk (H.K.K.); baoyuanye@gmail.com (Y.B.); 2 School of Creative Media, City University of Hong Kong, 83 Tat Chee Avenue, Hong Kong, China; 3 Centre for Robotics and Automation, City University of Hong Kong, 83 Tat Chee Avenue, Hong Kong, China; 4 CityU Shenzhen Research Institute, 8 Yuexing 1st Road, Shenzhen Hi-Tech Industrial Park, Nanshan District, Shenzhen 518057, China

**Keywords:** cell chirality, left–right asymmetry, stiffness, skeletal muscle myoblast, micropatterning, cell orientation

## Abstract

Reconstitution of tissue morphology with inherent left–right (LR) asymmetry is essential for tissue/organ functions. For skeletal muscle, the largest tissue in mammalian organisms, successful myogenesis requires the regulation of the LR asymmetry to form the appropriate muscle alignment. However, the key factor for reproducing the LR asymmetry of skeletal tissues in a controllable, engineering context remains largely unknown. Recent reports indicate that cell chirality may underlie the LR development in tissue morphogenesis. Here, we report that a rigid substrate is required for the chirality of skeletal muscle cells. By using alternating micropatterned cell-adherent and cell-repellent stripes on a rigid substrate, we found that C2C12 skeletal muscle myoblasts exhibited a unidirectional tilted orientation with respect to the stripe boundary. Importantly, such chiral orientation was reduced when soft substrates were used instead. In addition, we demonstrated the key role of actin stress fibers in the formation of the chiral orientation. This study reveals that a rigid substrate is required for the chiral pattern of myoblasts, paving the way for reconstructing damaged muscle tissue with inherent LR asymmetry in the future.

## 1. Introduction

Left–right (LR) asymmetry in tissue morphology is commonly seen in many organisms, such as climbing plants [1], and in the visceral distribution of Drosophila [2] and mammals [3]. The LR asymmetry pattern is closely connected with many tissue or organ functions. For instance, cardiac LR asymmetry mutants of zebrafish give rise to defects in jogging and cardiac looping [4]. Abnormal LR asymmetry development may also cause prenatal diseases and malfunction of organs [5]. As such, it is of vital importance to understand the development of LR asymmetry in tissue morphogenesis.

Skeletal muscle is the largest tissue in mammalian organisms. The appropriate function of skeletal muscle is to provide physical support essential for the metabolism of individuals [6,7]. When regenerating skeletal muscle in vitro, myoblasts should differentiate and form well-aligned myotubes as aligned muscle bundles in vivo [8], which requires the expression of the LR asymmetry to determine the specific orientation of the skeletal muscle cells. Recently, chirality in single cells was found essential for LR morphogenesis in tissue-level architecture [5], suggesting that cell chirality may be the ultimate cause to give rise to LR patterns in organisms. Thus, to rebuild skeletal muscle in a controllable, engineering context, it is essential to understand how and what factors regulate cell chirality in this process. Extensive studies have been conducted to demonstrate the epigenetic regulation during the myogenesis, such as DNA methylation in regulating the associated genes [3]. However, few studies have shown the biophysical properties in regulating the chiral pattern in skeletal myogenesis.

Substrate stiffness, as a key biophysical property, is closely related to intracellular stress fibers and focal adhesions [9,10]. Moreover, as actin stress fibers play important roles in cell mechanosensing [9,11], substrate stiffness may be an effective factor in guiding cell behaviors, such as cell migration [12] and differentiation [13,14]. Previously, we demonstrated that substrate rigidity can regulate the chiral orientation of NIH-3T3 fibroblasts [15]. However, different from fibroblasts, the chiral orientation of myoblasts has a more apparent role in tissue function, such as the direction of muscle contractility [8]. Here, using alternating micropatterned stripes of cell-adherent and cell-repellent regions on substrates with varied stiffness, we analyzed the chirality of C2C12 skeletal muscle myoblasts, based on the cell orientation with respect to the stripe boundary. Furthermore, by applying fluorescence staining and inhibitors of actin, we investigated the role of actin stress fibers in such chiral orientation. This study reveals the role of substrate stiffness as a biophysical factor in the development of the chirality of myoblasts, paving ways for the reconstruction and regeneration of muscle tissue.

## 2. Materials and Methods

### 2.1. Cell Culture and Treatment

C2C12 mouse myoblasts were cultured in Dulbecco’s Modified Eagle Medium (4 mM l-glutamine, 4500 mg/L glucose, 1 mM sodium pyruvate, and 1.5 g/L sodium bicarbonate), supplemented with 10% fetal bovine serum and 1% penicillin–streptomycin. Cells were kept at 37 °C in a humidified incubator (5% CO_2_ and 95% air) and were passaged every 2 days. For the experiment with the actin inhibitor, 1 μM of Cytochalasin D (Life Technology, Carlsbad, CA, USA) and 10 μM of Y27632 (Alexis, Farmingdale, NY, USA) were applied.

### 2.2. Micropatterning on Substrates with Varied Stiffness

Photolithography was applied to create cell-adherent stripes with a width of 200 μm or circles with an area of 3000 μm^2^ on substrates with varied stiffness [16]. For the ‘soft substrate’, 0.94 mL of polydimethylsiloxane (PDMS; Dow Corning, Midland, MI, USA) was evenly smeared on the pre-cleaned slides, followed by 5 h of standing and 70 °C baking overnight to form a 500-μm layer. The stiffness of the PDMS substrate was tuned by changing the ratio of elastomer to cross-linker, namely to 3:1, 10:1, and 30:1. After curing, the PDMS was treated for 2 min with air plasma (800 mTorr, 30 W) and coated with hexamethyldisilazane (HMDS, Sigma, St. Louis, MO, USA). Afterwards, AZ5214 photoresist (PR, AZ Electronic Materials, Luxembourg) was spin-coated on the HMDS/PDMS substrate at 2500 rpm and baked for 2 min at 95 °C. The substrate was then exposed under ultraviolet (UV) light (5 mW/cm^2^) for 80 s and developed using the developer (AZ400K:deionized water = 1:2) for 12 s to clean the exposed PR. Next, a 2-min air plasma (800 mTorr, 30 W) and HDMS coating was applied to the slides, and 10 μg/mL fibronectin solution (Life Technology, Carlsbad, CA, USA) was used to coat the exposed region for 30 min. The rest of the PR was removed by immersing in absolute ethanol on an orbital shaker for 15 min for three times. After being coated with 2% pluronic F127 (Sigma, St. Louis, MO, USA) in deionized water for 50 min, the chips were ready for cell seeding. For the “rigid substrate”, glass slides were first treated using a piranha solution (sulfuric acid to hydrogen peroxide = 3:1) for 30 min. HMDS was applied on the slides, and the following photolithography procedures and fibronectin/pluronic coating remained the same as in the preparation for the soft substrates.

### 2.3. Nano-Indentation Measurement

The elastic constants of the glass and of the PDMS substrate were measured by nano-indentation (Hysitron TI 950 TriboIndenter, Hysitron, Minneapolis, MN, USA). Testing samples were cut from the glass slides after the micropatterning. The elastic modulus ($E_{s}$) was calculated by $\frac{1}{E_{r}}=\frac{1-v_{i}^{2}}{E_{i}}+\frac{1-v_{s}^{2}}{E_{s}}$, where $E_{i}$ is the Young’s modulus of the indenter tip (1140 GPa), $v_{i}$ is the Poisson’s ratio of the indenter material (0.07), and $v_{s}$ is the Poisson’s ratio of the testing sample (0.3 for glass and 0.5 for PDMS). The reduced modulus ($E_{r}$) was obtained by the load–depth curve via the Nano Indenter, where $E_{r}=\frac{1}{2}\sqrt{\frac{\pi }{A}}\frac{dP}{dH}$, *A* is the residual indentation area, d*P* is the maximum loading force, and d*H* is the depth of penetration obtained by the unloading curve close to maximum load. Of note, different loading forces (set as 500.0, 40.0, or 15.0 μN) were adapted according to the sample’s thickness and elastic modulus level.

### 2.4. Fluorescence Staining and Imaging

To stain the cell nucleus, cells were seeded in the micropatterned chips with a density of 50,000 cells/cm^2^ for 30 min. The chips were then rinsed with DPBS to remove the unattached cells. The cells were immediately fixed for counting the initial cell density or cultured in growth medium for 12 h. After culture, the cells were treated with 4% paraformaldehyde (PFA) for 15 min, 0.1% Triton X-100 for 10 min, and 4′,6-diamidino-2-phenylindole (DAPI, 300 nM, Thermal Fisher Scientific, Waltham, MA, USA) staining for 5 min. To stain the actin filaments, the cells were seeded in circular micropatterns with a density of 5000 cells/cm^2^ for 3 h. Then, 4% PFA was applied to the chips for 15 min, 0.1% Triton X-100 for 10 min, Image-iT FX signal enhancer (Thermo Fisher Scientific, Waltham, MA, USA) for 30 min, and Rhodamine Phalloidin (1/40, Life Technology, Carlsbad, CA, USA) for 1 h. Before each step, the chips were rinsed with DPBS. Afterwards, the chips were mounted with Fluoromount G (Electron Microscopy Sciences, Inc., Hatfield, PA, USA). The images were acquired using an inverted fluorescence microscope (Nikon, Tokyo, Japan).

### 2.5. Analysis of Cell Orientation and Density

**Figure 1:**
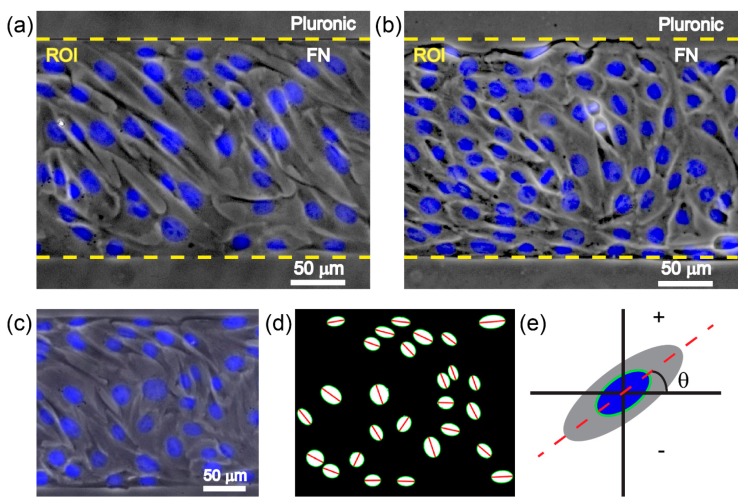
Schematic showing the definition of cell orientation in micropatterned stripes. (**a**,**b**) On a rigid substrate (**a**, glass) or a soft substrate (**b**, polydimethylsiloxane (PDMS), ratio of elastomer to cross-linker = 10:1), phase contrast microscopy showing C2C12 cells with 4′,6-diamidino-2-phenylindole (DAPI) nuclei staining cultured in micropatterned fibronectin (FN) stripes spaced by pluronic stripes; (**c**) phase contrast microscopy of C2C12 cells with DAPI nuclei staining, demonstrating that the long axis of the cell nucleus is aligned with the long axis of the spindle-shaped cell; (**d**) processed image of DAPI nuclei staining showing cell orientations. The green ellipse boundary shows the outline of the cell nuclei, and the red line shows the long axis of the cell nuclei; (**e**) the angle *θ* between the long axis of the cell nuclei and the horizontal axis aligned with the stripe boundary, which is defined as a positive value when the acute angle *θ* is within [0, 90] and as a negative value when the acute angle *θ* is within [−90, 0]. By counting the percentage of negatively and positively oriented cells in each stripe, the chirality is revealed when the difference between those percentages from multiple stripes is statistically significant.

The region of interest (ROI), i.e., the cells inside the micropatterned fibronectin stripes (Figure 1a,b), was cropped and analyzed using a MATLAB program. The cell orientation was analyzed based on an angular alignment *θ* between the long axis of the cell nucleus and the horizontal axis aligned with the stripe boundary (Figure 1c–e). A MATLAB program was applied to conduct nucleus segmentation and measurement of the angle *θ*, as described previously [15]. In brief, a Gaussian filter and a threshold were used to convert the raw fluorescence microscopy images into binary images. After isolating the individual nucleus from the clusters, the orientation of the long axis was obtained by ellipse detection (Figure 1d). The acute angle *θ* is defined as negatively oriented when the acute angle *θ* is within [−90, 0] and as positively oriented when *θ* is within [0, 90] (Figure 1e). To analyze the chirality, we counted the percentage of the positively and negatively aligned cells within one stripe. By collecting the percentages from multiple stripes, the evident chirality can be shown by the significant difference between the two percentages. Furthermore, to calculate the cell density, we used the total cell counts of each stripe divided by the stripe area.

### 2.6. Statistical Analysis

The Student’s *t*-test was applied to compare the bias between the percentage of cells with negative and positive orientation. The confidence level was set to be 0.05 for all statistical tests. The statistical significance was symbolized by ns (*p* > 0.05), * (*p* ≤ 0.05), ** (*p* value ≤ 0.01), *** (*p* value ≤ 0.001), or **** (*p* value ≤ 0.0001).

## 3. Result

### 3.1. Measurement of Substrate Stiffness

**Figure 2:**
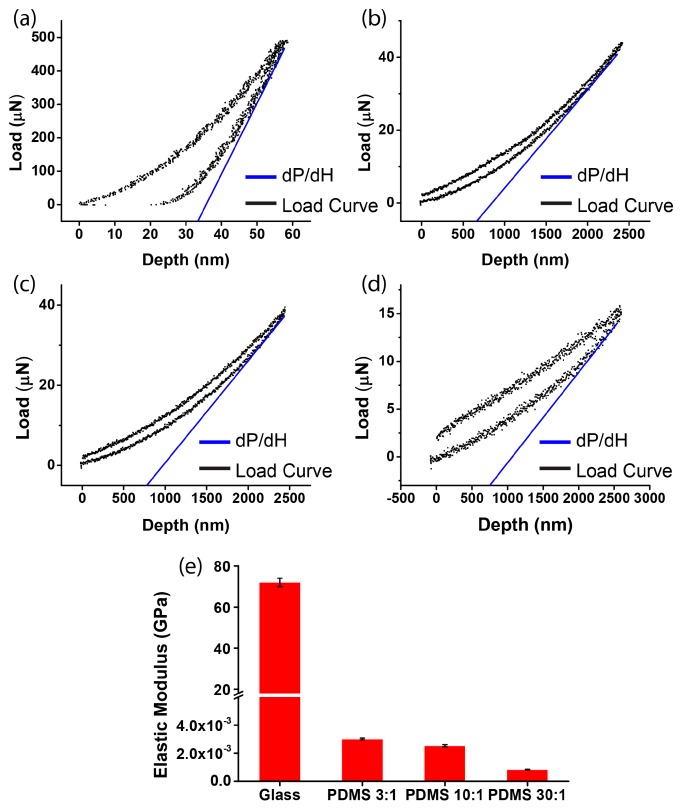
Measurement of substrate stiffness by nano-indentation. The load–depth curve of the micropatterned substrates of (**a**) glass (d*P* was 490.7 μN and d*H* was 58.7 nm); (**b**) PDMS, ratio of elastomer to cross-linker = 3:1 (d*P* was 43.9 μN and d*H* was 2420.3 nm); (**c**) PDMS, ratio of elastomer to cross-linker = 10:1 (d*P* was 38.7 μN and d*H* was 2447.8 nm); and (**d**) PDMS, ratio of elastomer to cross-linker = 30:1 (d*P* was 15.2 μN and d*H* was 2599.8 nm); (**e**) the elastic modulus of the four substrates after repeated experiments (mean ± SD; *n* = 3).

We first measured the stiffness of different substrates. To obtain the elastic modulus of the glass and the PDMS substrates (ratio of elastomer to cross-linker = 3:1, 10:1, or 30:1), the reduced Young’s modulus *E*_r_ was derived by nano-indentation (Figure 2a–d). After calculation, the elastic moduli of the four substrates were obtained as 71.70 ± 1.58 GPa (glass), 3.02 ± 0.068 MPa (PDMS, ratio of elastomer to cross-linker = 3:1), 2.53 ± 0.078 MPa (PDMS, ratio of elastomer to cross-linker = 10:1), and 0.835 ± 0.068 MPa (PDMS, ratio of elastomer to cross-linker = 30:1). For the soft substrate, the elastic modulus of the PDMS substrates can be tuned, as it decreased with the increase of the ratio of elastomer to cross-linker (Figure 2e). Moreover, since the elastic modulus of glass is more than 10,000 times greater than that of the PDMS substrates, the glass substrate was regarded as being a rigid substrate.

### 3.2. Chirality of Cell Orientation

**Figure 3:**
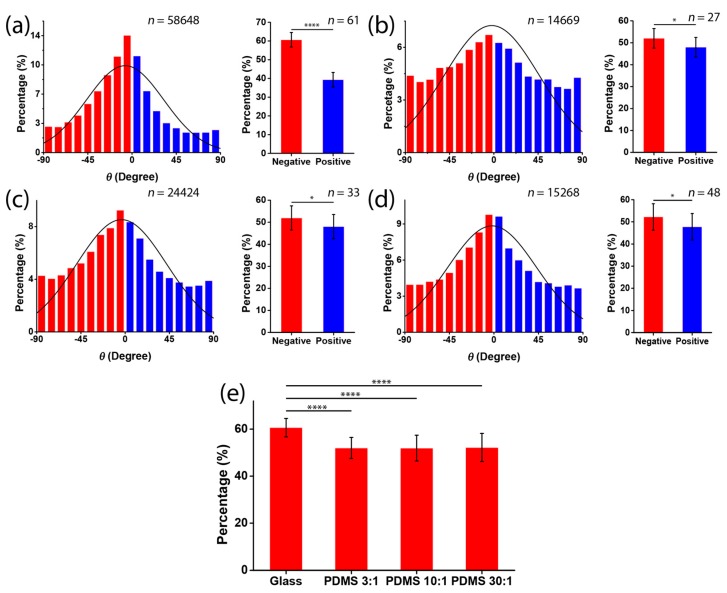
Chirality of cell orientation on substrates with different stiffness. (**a**–**d**) Histograms of cell orientation *θ* when cells were cultured in micropatterned stripes fabricated on glass substrates (**a**); PDMS substrate, ratio of elastomer to cross-linker = 3:1 (**b**); PDMS substrate, ratio of elastomer to cross-linker = 10:1 (**c**); and PDMS substrate, ratio of elastomer to cross-linker = 30:1 (**d**); (**e**) the percentages of negatively aligned cells on the four different substrates (mean ± SD).

Next, we investigated the chirality of cell orientation in micropatterned stripes with varied substrate stiffness. C2C12 cells were seeded on substrates containing alternating micropatterned stripes of cell-adherent fibronectin and cell-repellent pluronic. After the cells had been seeded for 12 h, the cells were stained by DAPI, such that the orientation angle of the nucleus with respect to the stripe boundary could be determined. Notably, the measurement of the cell nucleus was based on the fact that the long axis of the cell nucleus was aligned with the long axis of the spindle-shaped cell (Figure 1a–c). When cultured on rigid substrates, the cell orientation was negatively biased (Figure 3a). This indicated that there was a strong chirality of C2C12 cells when they were cultured on rigid substrates. As for the soft substrates, the average orientation angles for PDMS 3:1, 10:1, and 30:1 were −2.14° ± 48.48°, −3.62° ± 45.68°, and −0.5° ± 49.42°, respectively (Figure 3b–d). In addition, the corresponding percentages of the negatively aligned cells showed that this chirality was greatly reduced on the PDMS substrate (Figure 3e), regardless of the ratio of elastomer to cross-linker, which may be due to the ratio of elastomer to cross-linker causing only a slight change in substrate stiffness (Figure 2e). Notably, on the soft substrate, the orientation angle converged to zero, demonstrating a preferred coherent alignment with the stripe boundary. It indicates that the soft substrate only suppressed the chirality but did not disorganize the cell alignment. Taken together, our results suggest that a rigid substrate is required for the expression of the chirality of cell orientation.

**Figure 4:**
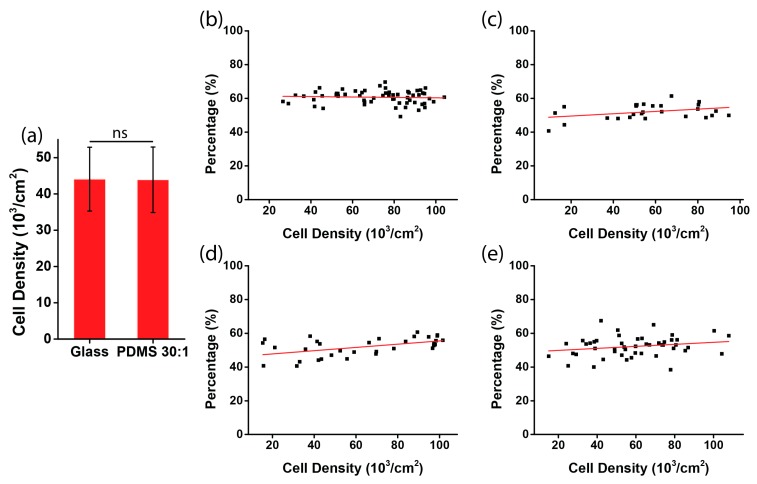
Measurement of cell density in the micropatterned stripes. (**a**) Initial cell density in the stripes on glass and on the PDMS substrate (ratio of elastomer to cross-linker = 30:1); (**b**–**e**) the percentages of negatively aligned cells with respect to cell density. The stripes were micropatterned on substrates of glass (**b**); PDMS, ratio of elastomer to cross-linker = 3:1 (**c**); PDMS, ratio of elastomer to cross-linker = 10:1 (**d**); and PDMS, ratio of elastomer to cross-linker = 30:1 (**e**).

To rule out a possible involvement of other factors, such as cell density or biocompatibility on different substrates, we examined the percentage of negatively oriented cells with respect to the cell density. The initial cell densities on glass and on the PDMS substrate were not statistically different (Figure 4a, *p* value = 0.95), which indicates that after cell attachment, the number of cells on the rigid and soft substrate were equivalent. After 12 h, for the four types of substrates, the cell densities were all within the range of 20,000–100,000 cells per cm^2^ (Figure 4b–e), indicating that a different substrate stiffness did not result in a difference of the cell plating density. Moreover, regarding the chirality with respect to the fluctuation of the cell density within the range, we found that the percentage of negatively aligned cells was independent of the cell density, as the fitting lines were all very horizontal (red lines in Figure 4b–e). Thus, it can be concluded that the dependence of the chiral pattern on substrate stiffness is not caused by other factors, such as cell density or biocompatibility, when a different substrate is used.

### 3.3. Role of Actin

**Figure 5:**
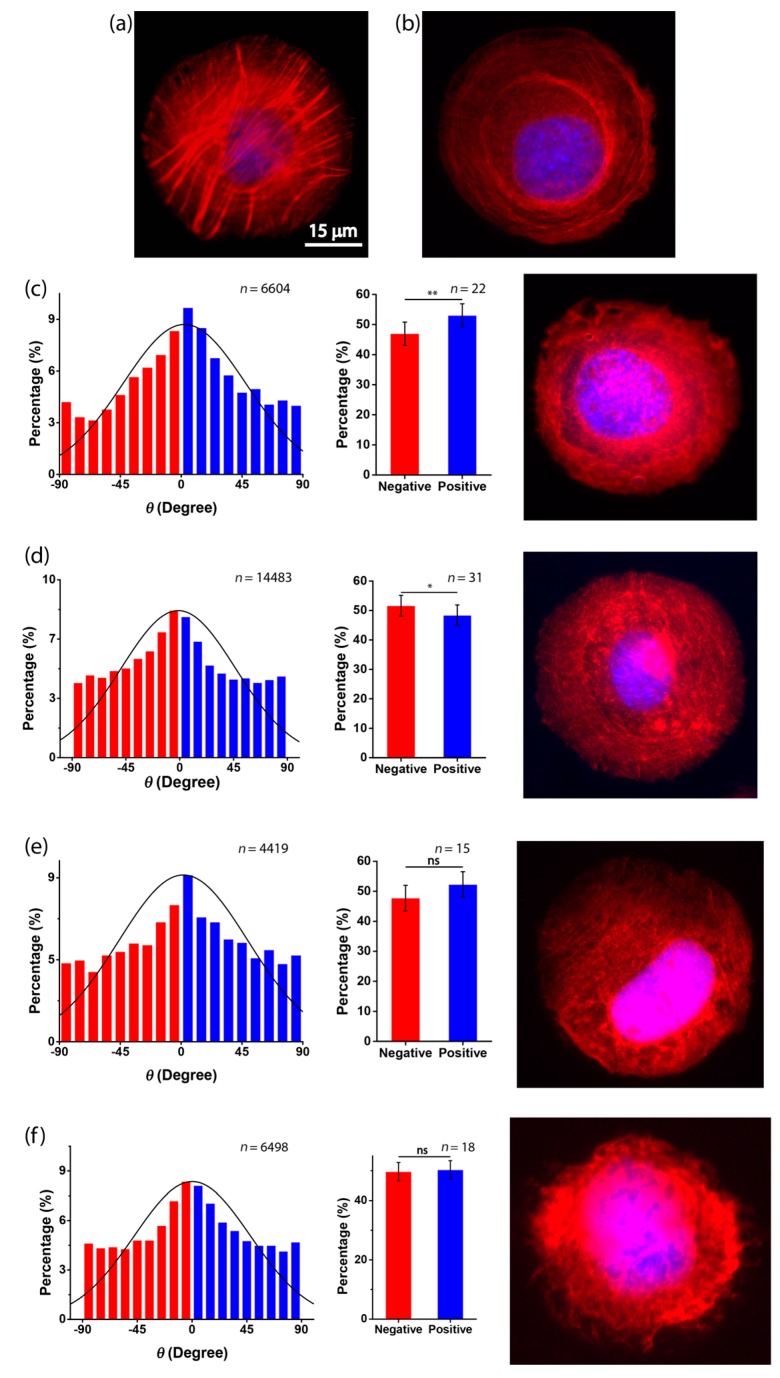
Assembly of actin filaments is required for cell chirality. (**a**,**b**) Fluorescence microscopy images of actin filaments of untreated C2C12 myoblasts cultured in circular micropatterns on a glass substrate (**a**) or a PDMS substrate (ratio of elastomer to cross-linker = 30:1) (**b**); (**c**,**d**) fluorescence microscopy images of actin filaments of C2C12 myoblasts cultured in circular micropatterns on a glass substrate and the histogram of cell orientation when cultured in micropatterned stripes on a glass substrate with the presence of Cytochalasin D (**c**) or Y27632 (**d**); (**e**,**f**) fluorescence microscopy images of actin filaments of C2C12 myoblasts cultured in circular micropatterns on a PDMS substrate (ratio of elastomer to cross-linker = 30:1) and the histogram of C2C12 orientation cultured in micropatterned stripes on a PDMS substrate (ratio of elastomer to cross-linker = 30:1) with the presence of Cytochalasin D (**e**) or Y27632 (**f**).

To further investigate the dependence of the cell chirality on substrate stiffness, we applied fluorescence staining and inhibitors of actin. The soft substrate was known to disorganize and down-regulate the formation of actin filaments [17]. Thus, the reduced chirality on the soft substrate may also be mediated by the disassembly of actin stress fibers. To visualize the actin filaments, we applied fluorescence staining of actin on different substrates. Circular micropatterns were used for the analysis of the actin cytoskeleton because they provide a geometric confinement of the cell shape that highlights the classification of the transverse arc and dorsal stress fibers of individual cells [7]. When cells were cultured in circular micropatterns on a rigid substrate, clear actin stress fibers can be observed (Figure 5a). However, these filaments were dissembled on the soft substrate (Figure 5b), suggesting that actin is involved in the dependence of chirality on substrate stiffness.

To further explore this possibility, two actin inhibitors—Cytochalasin D, that destroys actin microfilaments by inhibiting actin polymerization, and Y27632, that inhibits Rho-associated kinase (ROCK)—were used to treat the C2C12 cells [5,15]. When cells were treated with Cytochalasin D and Y27632 on a rigid substrate, the actin stress fibers were damaged (Figure 5c,d). For the chirality of cell orientation, the results showed that when Cytochalasin D was applied to the C2C12 cells on rigid substrates (Figure 5c), the percentage of negatively aligned cells became 46.95% ± 3.84%, which was greatly reduced as compared to the untreated cases (60.7% ± 3.90%) (Figure 3a). Interestingly, the cell chirality was even reversed when Cytochalasin D was present, which is consistent with previous findings [11]. Additionally, when Y27632 was applied to the C2C12 cells cultured on a rigid substrate, we found that the cells were still more negatively aligned, but the statistical significance of the chirality was reduced (Figure 5d), demonstrating the abrogation of chirality when the actin inhibitors were present. Moreover, when combining the soft substrate and actin inhibition (Cytochalasin D or Y27632), we found that the actin filaments of the cells were again dissembled, and the chirality was completely neutralized (Figure 5e,f). Taken together, our results suggest that the dependence of the chiral pattern on substrate stiffness could be associated with the assembly of actin filaments.

## 4. Discussion

The dependence of the chiral orientation of skeletal muscle tissue has rarely been addressed previously. Here, by culturing C2C12 mouse muscle myoblasts in micropatterned stripes with varied substrate stiffness, we found that C2C12 cells oriented with a specific chiral pattern when cultured on rigid substrates and that such chirality was neutralized when they were cultured on soft substrates. To our best knowledge, it is the first demonstration showing that the exhibition of the chiral pattern of muscle myoblasts is dependent on the substrate stiffness, and this finding is consistent with our previous results that substrate rigidity provides an effective biophysical factor to regulate the chirality of NIH-3T3 fibroblasts [15].

However, different from NIH-3T3 fibroblasts, myoblasts are the fundamental units of muscle tissue, and the orientation and intercellular alignment are known to be important for the fusion and differentiation in myotube formation [8]. Since the elastic modulus of trabecular tissue and articular cartilage is in the range of GPa and MPa, respectively, our results imply that the requirement of a rigid substrate for the expression of myoblast chirality may be related to a mechanical interface between skeletal muscle tissue and other rigid tissues, such as trabecular tissue or articular cartilage. We speculate that once the chiral pattern initiates, it may be integrated and may regulate cell alignment through the propagation of cell–cell contacts. Such interactions subsequently lead to an amplified, specific LR bias in the alignment of myotubes, eventually guiding the LR asymmetry features in tissue morphogenesis. As a potential mechanism that regulates muscle layer formation and function, e.g., the direction of muscle contractility, our finding provides greater insights for guiding the orientation of muscle tissue for tissue reconstruction.

Moreover, we further demonstrated that actin stress fibers play a vital role in the formation of a chiral pattern of C2C12 cells. Actin stress fibers can generate a contractile force, which controls the formation of focal adhesion [7,9,15]. Meanwhile, actin stress fibers can be regulated by the substrate stiffness, which is also responsible for cell mechanosensing. Therefore, by destroying actin stress fibers, the cell mechanosensing is disrupted, which in turns suppresses the chirality. Thus, our results indicate that chirality could be a behavior of cell mechanosensing, suggesting the importance of constituting an adequate mechanical environment for the accurate reproduction of the LR bias in tissue morphogenesis.

Interestingly, compared to Y27632, that only suppressed the chirality, Cytochalasin D can reverse the chiral pattern when cells were cultured on rigid substrates. However, the mechanism of how the treatment of Cytochalasin D can reverse the chiral orientation remains unclear. In fact, cell chirality can be bidirectional, depending on the expression level of α-actinin-1 [18]. As the treatment of Cytochalasin D could induce the synthesis of α-actinin for skeletal muscle cells [19], the increased expression of α-actinin could be the underlying mechanism for the reversal of the cell chirality. Interestingly, such reversal is not observed in other cells types, such as 3T3 fibroblasts [7,15], suggesting that the increased synthesis of α-actinin by Cytochalasin D could be specific to skeletal muscle cells.

## 5. Conclusions

In summary, this paper reveals that C2C12 mouse muscle myoblasts exhibit a chiral orientation on rigid substrates, but not on soft substrates. The chirality is closely related to the formation of actin stress fibers. Through this work, the role of substrate stiffness in the chirality of skeletal muscle cells was revealed, providing a potential approach for guiding the LR direction of skeletal muscle tissue in the future.
